# Molecular Mechanisms Underlying the Establishment, Maintenance, and Removal of DNA Methylation in Plants

**DOI:** 10.1146/annurev-arplant-083123-054357

**Published:** 2025-03-03

**Authors:** Guohui Xie, Xuan Du, Hongmiao Hu, Jiamu Du

**Affiliations:** 1 Shenzhen Key Laboratory of Plant Genetic Engineering and Molecular Design, Institute of Plant and Food Science, Department of Biology, School of Life Sciences, Southern University of Science and Technology, Shenzhen, China; 2 Guangdong Provincial Key Laboratory for Plant Epigenetics, College of Life Sciences and Oceanography, Shenzhen University, Shenzhen, China; 3 MRC Laboratory of Molecular Biology, Cambridge, United Kingdom; 4 Institute for Biological Electron Microscopy, Southern University of Science and Technology, Shenzhen, China

**Keywords:** epigenetic regulation, RNA-directed DNA methylation, small interfering RNA, long noncoding RNA, histone modification, DNA demethylation

## Abstract

Methylation at the fifth position of the cytosine base (5mC) is a critical DNA modification with important functions in gene silencing, genome imprinting, and suppression of transposable elements in eukaryotes. Bio-chemically, DNA methylation is dynamically regulated by three critical processes: the de novo establishment of DNA methylation, the maintenance of DNA methylation by preexisting methylation patterns, and the removal of DNA methylation. In plants, DNA methylation is very complex with unique features. In past decades, a series of biochemical and structural studies, especially empowered by the recent breakthroughs of high-resolution cryogenic electron microscopy, have helped uncover the molecular mechanisms underlying the establishment, maintenance, and removal of DNA methylation in plants. This review summarizes recent research advances in these three aspects of DNA methylation and lays out a molecular view of plant DNA methylation from biochemical and structural perspectives.

## 1 Introduction

Methylation at the fifth position of the cytosine base of DNA [5-methylcytosine (5mC)] is a critical epigenetic modification in mammals and plants that plays essential roles in gene regulation, genome defense, gene imprinting, and suppression of transposable elements (TEs) and repetitive sequences (68, 150). Whereas mammalian genomes are predominately methylated at symmetric CG sites, in the genome of the model plant *Arabidopsis thaliana*, high-level DNA methylation happens in three sequence contexts: CG, CHG (H meaning A, T, or C), and CHH, representing a much more complex system (17, 82). In plants, the TE-enriched pericentromeric heterochromatin and some small euchromatin patches are rich in DNA methylation in all three sequence contexts, which serves to silence the TEs and maintain genome integrity (17, 68, 82). CG methylation is also enriched at the exons of some genes, a phenomenon termed gene body methylation (gbM) (17, 82). gbM is evolutionarily conserved and appears to be associated with housekeeping genes with moderate expression levels, although the underlying regulatory mechanism and functional significance are debated (7).

Biochemically, the dynamic regulation of DNA methylation consists of multiple steps (68). First, DNA methylation can be de novo established to create the initial methylation pattern. Second, during DNA replication, the daughter strand is initially unmethylated, which requires the recovery of DNA methylation guided by the preexisting pattern on the mother strand, termed the maintenance of DNA methylation. Third, DNA methylation marks from actively transcribed genes are removed to balance DNA methylation and prevent abnormal over-silencing, termed DNA demethylation. In plants, these steps are guided by distinct pathways involving different sets of biochemical reactions specific to each of the three methylation sequence contexts that dynamically and precisely regulate DNA methylation, shaping a complex network. In the past few decades, dozens of plant genes have been identified as having critical roles in DNA methylation (68, 94, 150) (Table 1). A series of structural and biochemical studies of these critical factors has revealed the underlying mechanisms of DNA methylation dynamics, offering a molecular image of the process (Table 1). Since 2013, the resolution revolution of cryogenic electron microscopy (cryo-EM) technology (16) has substantially promoted and accelerated mechanistic studies into DNA methylation in plants, yielding several landmark findings that deepened our understanding of DNA methylation in plants. Several excellent reviews have already summarized the component identification, regulation, and functions of DNA methylation in plants (68, 93, 94, 148, 150). In this review, we focus on the biochemical mechanisms underlying the establishment, maintenance, and removal of DNA methylation in plants to draw a comprehensive molecular picture of these biological events.

## 2 Establishment of DNA Methylation in Plants

### 2.1 Overview of RNA-Directed DNA Methylation

**Figure 1:**
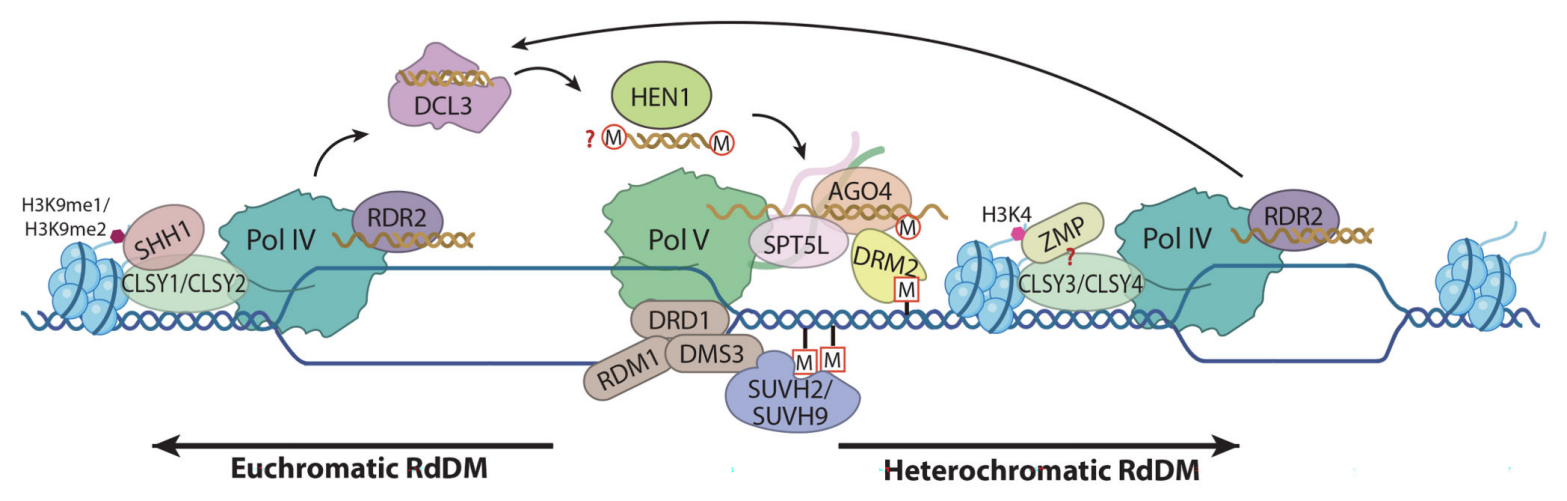
Establishment of DNA methylation in plants by RdDM. In euchromatin and heterochromatin, CLSY1/CLSY2 and CLSY3/CLSY4, respectively, direct the Pol IV–RDR2 complex to produce a double-stranded precursor siRNA. This step may be guided by the histone readers SHH1 and ZMP, respectively. These precursor siRNAs are then diced by DCL3 to produce a 23–24-nt siRNA duplex that is methylated by HEN1, with the 24-nt strand being loaded into AGO4 as the guide strand. At the same time, Pol V is recruited to the methylated DNA region by the chromatin remodeling complex DDR (DRD1–DMS3–RDM1) bound to SUVH2/SUVH9. Pol V, Pol V–produced lncRNA, and Pol V–associated SPT5L together recruit AGO4 by protein–protein interactions and RNA base-pairing to trigger DNA methylation via DRM2. Whether the 23-nt RNA is methylated and whether ZMP associates with CLSY3/CLSY4 remains a subject of debate; these two steps are thus indicated with question marks. Abbreviations: AGO4, ARGONAUTE 4; CLSY, CLASSY; DCL3, DICER-LIKE 3; DMS3, DEFECTIVE IN MERISTEM SILENCING 3; DRD1, DEFECTIVE IN RNA-DIRECTED DNA METHYLATION 1; DRM2, DOMAINS REARRANGED METHYLTRANSFERASE 2; H3K4, histone 3 lysine 4; H3K9me, histone 3 lysine 9 methylation; HEN1, HUA ENHANCER 1; M, methylation; Pol, RNA polymerase; RdDM, RNA-directed DNA methylation; RDM1, RNA-DIRECTED DNA METHYLATION 1; RDR2, RNA-DEPENDENT RNA POLYMERASE 2; SHH1, SAWADEE HOMEODOMAIN HOMOLOG 1; siRNA, small interfering RNA; SPT5L, SUPPRESSOR OF TY INSERTION 5-LIKE; SUVH, SU(VAR)3–9 HOMOLOG; ZMP, ZINC FINGER, MOUSE DOUBLE-MINUTE/SWITCHING COMPLEX B, PLUS-3 PROTEIN.

The establishment of DNA methylation in plants relies on the plant-specific RNA-directed DNA methylation (RdDM) pathway (68, 93, 94). Similar to the widespread small RNA (sRNA)-induced chromatin silencing pathways in fungi and animals, plant RdDM requires a long RNA transcript and a complementary sRNA to base-pair and mediate DNA methylation, establishing chromatin silencing (44). Whereas animals and fungi employ RNA polymerase II (Pol II) to produce both the sRNA precursor and the matching long RNA transcript, plants have evolved the specialized DNA-dependent RNA polymerases (DdRPs) Pol IV and Pol V to synthesize the small interfering RNA (siRNA) precursor and long noncoding RNA (lncRNA), respectively (44, 93, 94). The canonical RdDM pathway is initiated by Pol IV transcription, with its transcripts undergoing stepwise processing by RNA-DEPENDENT RNA POLYMERASE 2 (RDR2) and DICER-LIKE 3 (DCL3) into 24-nucleotide (nt) siRNAs before they are loaded into ARGONAUTE 4 (AGO4) (or its paralogs AGO6 and AGO9) (43, 60, 99, 108, 161, 162). Pol V produces scaffold lncRNA, and together they serve as a platform to recruit the AGO4–siRNA complex to trigger DNA methylation by DOMAINS REARRANGED METHYLTRANSFERASE 2 (DRM2) (11, 12, 108, 135, 136) (Figure 1; Table 1). Additionally, several alternative pathways have been described that also achieve RdDM in certain conditions. For instance, RDR6 can process some Pol II transcripts into double-stranded RNA (dsRNA), which is then diced into 21-nt siRNA by DCL1 and subsequently loaded into AGO4 or AGO6 for guiding Pol V–mediated RdDM (139). In rice (*Oryza sativa*), some Pol II–derived hairpin RNAs can be processed by DCL3 to produce 24-nt long microRNAs that are then loaded into AGO4 to establish RdDM (140). In addition, the Pol II–RDR6–DCL4/DCL2–AGO2 module mirrors the Pol IV–RDR2–DCL3–AGO4 module of canonical RdDM to produce the AGO2–siRNA complex, which further interacts with the Pol V–lncRNA complex to establish RdDM at new TEs (98, 107). Generally, the canonical and noncanonical RdDM pathways follow similar biochemical routes as the Pol–RDR–DCL–AGO module to generate sRNA to further interact with Pol V–lncRNA for initiating DNA methylation. We will focus only on the canonical RdDM pathway in this review.

### 2.2 The Mechanism of 24-Nucleotide Small Interfering RNA Biogenesis

Although there are multiple types of siRNAs and DdRPs in plants, only the 24-nt siRNA exclusively produced by the Pol IV–RDR2–DCL3 module is specifically selected for the canonical RdDM. To guarantee this specificity, Pol IV, RDR2, and DCL3 should therefore relay their product in succession. Like Pol II, Pol IV mostly prefers to initiate transcription with a 5′ purine, especially A, and to a lesser extent G, which is preceded by a pyrimidine ahead of the initiation site in vivo; this preference for transcription initiation site is also observed in vitro (9, 117, 147), suggesting an intrinsic biochemical tendency of Pol IV to start transcription with a 5′ A. So far, no Pol IV transcription initiation factors have been identified; thus, the precise initiation mechanism is still unclear. Pol IV transcripts lack the standard features of Pol II transcripts, such as a 5′ cap, a 3′ poly(A) tail, and intron splicing, but do exhibit a 5′ monophosphate, suggesting that an as-yet-unidentified phosphatase exists or that Pol IV possesses intrinsic phosphatase activity to dephosphorylate the 5′ end of the nascent RNA (9, 75, 147). At the 3′ ends of transcripts, one or two nontemplated nucleotides are always incorporated into Pol IV transcripts, with this 3′ misincorporation always associated with transcription over 5mC in the template DNA (9, 147). Moreover, Pol IV transcripts are unexpectedly short, only ∼26–45 nt in length, with a peak at around 30 nt, which would be sufficient for only one 24-nt siRNA to be produced; this led to the hypothesis of the one precursor–one siRNA model (9, 89, 147). In vitro, Pol IV shows unambiguous transcriptional activity but with a lower rate and lower fidelity than Pol II (39, 89, 117).

The Pol IV transcripts are not directly cut into 24-nt fragments. Rather, they serve as templates for RDR2 to synthesize dsRNA, which is only then precisely diced by DCL3 to 24 nt in length. Overall, the Pol IV, but not the RDR2, transcripts mostly possess the same sequences as the mature 24-nt siRNAs, suggesting that Pol IV RNAs are primarily selected as the guide strands for 24-nt siRNAs (9, 147). Although Pol IV and RDR2 independently exhibit transcription activity in vitro, they physically associate, and their RNA synthesis activities are coupled with extensive crosstalk (39, 46, 117). Using single-stranded DNA (ssDNA) as a template, the Pol IV–RDR2 complex produces only ssRNA transcripts but no dsRNA products (117). Annealing a nontemplate strand DNA to the template ssDNA leads to Pol IV arresting and shorter Pol IV transcripts (117). This can further initiate the synthesis of the second strand RNA by RDR2 to yield a dsRNA product, suggesting an essential role for nontemplate strand DNA in dsRNA production by Pol IV–RDR2 (117). However, the Pol IV–RDR2 complex could transcribe the bacteriophage M13 ssDNA in vitro to produce dsRNA de novo, probably because the M13 ssDNA possesses both the ssDNA regions for Pol IV initiation and extensive self-annealed dsDNA regions for second strand synthesis by RDR2 (117), providing an ideal artificial system to trace the strand-biased features. In this assay, Pol IV and RDR2 transcripts ranged in length from 30–50 nt with a peak around 30 nt (117). It is interesting that more Pol IV transcripts than RDR2 transcripts are shorter than 30 nt in this assay, implying a ∼30-nt length criterion for coupling the second strand synthesis by RDR2 to Pol IV (117). RDR2 bypasses the first one or two nucleotides from the 3′ end of Pol IV RNA, initiates transcription with a 5′ triphosphorylated nucleotide, and shows a terminal nucleotide transferase activity, adding one nontemplated nucleotide to the 3′ end of its transcripts (9, 30, 117). Therefore, Pol IV–RDR2 produces short dsRNAs of ∼26–45 base pairs (bp) in length and with molecular signatures of a 5′ monophosphorylated A (Pol IV strand) together with a 1-nt nontemplated 3′ overhang (RDR2 strand) at one end and a 1- or 2-nt 3′ overhang (Pol IV strand) with a 5′ triphosphate (RDR2 strand) at the other end.

Cryo-EM structures of the Pol IV–RDR2 complex have helped elucidate their unique enzymatic behaviors and coupling mechanism (22, 30, 46). The two largest subunits of eukaryotic DdRPs directly contribute to transcription. The largest subunit of Pol IV, NUCLEAR RNA POLYMERASE D 1 (NRPD1), is unique among DdRPs, while its second largest subunit, NRPD2, is shared with Pol V but not with Pol II (112). The Pol IV structure resembles that of other classic eukaryotic DdRPs, such as Pol II (18, 46). RDR2 adopts a closed ring-like structure with an active site resembling that of multisubunit DdRPs, despite being a single polypeptide (18, 22, 30, 46), suggesting a shared primary RNA polymerization reaction mechanism. In the absence of RNA, RDR2 adopts a closed confirmation that only permits template ssRNA to approach the active center; importantly, RDR2 has a narrow exit channel in this closed conformation that prohibits the release of the dsRNA product (22, 30, 46). A significant conformational change occurs upon the addition of RNA to RDR2, leading to the opening of the exit channel, which allows the dsRNA product to go through (22). To transfer the nascent 3′ end but not the preexisting 5′ end of Pol IV RNA from Pol IV to RDR2 for initiating dsRNA synthesis, RDR2 docks with the secondary channel of Pol IV, thus connecting the Pol IV secondary channel to the template entrance channel of RDR2 (46). Consequently, Pol IV RNAs can move bidirectionally, either in a forward release mechanism through the canonical RNA exit channel or through a backtracking mechanism into the Pol IV secondary channel to approach the active center of RDR2. In the backtracking conformation, the 3′ end of the nascent Pol IV RNA moves from the Pol IV active center to successively pass through the secondary channel of Pol IV and the entrance channel of RDR2, which can together accommodate about 20-nt RNA, allowing the RNA to approach the RDR2 active center for initiating dsRNA synthesis (46).

Further cryo-EM structures of Pol IV elongation complexes of a nucleotide addition cycle reveal that the trigger loop, a Pol IV catalytic element, has fewer interactions with substrate NTP compared to those of Pol II, partially explaining its low fidelity and lower efficiency in NTP incorporation (26). Biochemically, Pol IV favors a pretranslocation state and thereby is less efficient in forward translocation but is effective in backtracking (26). Pol IV NRPD2 and Pol V NRPE2 are in fact the same polypeptide, encoded by the same gene (112). Therefore, we hypothesize that the Pol IV NRPD2 may function in a similar way as NRPE2, which arrests transcription elongation and promotes backtracking through interactions with the non-template strand DNA of the transcription bubble (112, 142) (for a detailed discussion about Pol V NRPE2, see Section 2.3). Indeed, the NRPD2 has extensive interactions with the non-template strand DNA in the Pol IV backtracking conformation structure, despite not being identical to NRPE2 in Pol V elongation conformation (26, 142). This may help explain the results of the abovementioned biochemical assay in which Pol IV–RDR2 produced ssRNA using a ssDNA template but dsRNA only upon the addition of a complementary DNA strand (117). Here, the complementary DNA strand may serve as an anchoring site for NRPD2 to promote backtracking and to feed the Pol IV RNA to RDR2 (117, 141). Moreover, a ∼10-nt RNA in an intact transcription bubble allowing the interactions between NRPD2 and nontemplate DNA and a ∼20-nt RNA connecting the active centers of Pol IV and RDR2 are both required to precisely and robustly transfer Pol IV RNA to the RDR2 active center. Given these findings, a dual measurement model for dsRNA production by Pol IV–RDR2 was proposed (141). The dual measurement hypothesis posits that the maximal efficiency of Pol IV–RDR2–produced dsRNA must be at least 30 nt, which fits well with the enrichment of Pol IV RNA at ∼30 nt long in vivo and with the ∼30-nt RNA length criterion to couple the synthesis of the first strand RNA by Pol IV and the second strand by RDR2 (9, 117, 141, 147). When Pol IV RNA is shorter than 30 nt, backtracking is insufficient and cannot precisely deliver the 3′ end of the Pol IV– produced RNA to RDR2 to trigger dsRNA synthesis. Consistently, more Pol IV strands but fewer RDR2 strands were observed in Pol IV–RDR2 transcripts shorter than 30 nt when using bacteriophage M13 DNA as a template in vitro (117, 141). When the Pol IV RNA is longer than 30 nt, it can fully occupy a whole transcription bubble (∼10 nt) and the interpolymerase channel connecting the active centers of Pol IV and RDR2 (∼20 nt). This would enable the full binding of the nontemplate strand DNA by NRPD2, which triggers strong backtracking to robustly feed the 3′ end of the Pol IV RNA to RDR2 and initiate synthesis of the RDR2 strand (46, 141). This in turn pulls the Pol IV RNA back from the Pol IV active site to trigger the dsRNA-induced termination of Pol IV transcription (46, 141). As NRPD2 constantly pushes the Pol IV RNA back upon elongation, RNAs with a minimum length of 30 nt, just long enough to meet the ∼30-nt RNA criterion, have the highest chance to efficiently backtrack and precisely approach the RDR2 active center, possibly explaining the ∼30-nt peak seen for the 26–45-nt Pol IV RNA (9, 117, 141, 147).

During backtracking, Pol IV barely interacts with the backtracked RNA and does not have the docking site for the RNA cleavage factor transcript elongation factor IIS (TFIIS) (26, 46). Therefore, Pol IV would probably bypass the backtrack-induced RNA cleavage, leading it to omit the RNA cleavage-mediated proofreading, which further explains the low fidelity of Pol IV transcription (89). Moreover, the backtracking of Pol IV RNA would bring Pol IV back to its original transcription initiation site, plausibly to trigger another round of transcription (46). Multiple cycles of Pol IV transcription followed by backtracking may thus amplify siRNA copies. Therefore, the fidelity of the sequence seems less critical, plausibly explaining the apparent tolerance to low Pol IV accuracy.

While neither Pol IV nor RDR2 has been reported to directly interact with DCL3, DCL3 was reported to colocalize in the same nuclear speckles with Pol V but not Pol IV, suggesting the potential diffusion of the precursor dsRNA released from the Pol IV–RDR2 complex to reach DCL3 in RNA-processing speckles rather than direct physical interaction and transfer (72, 106). DCL3 prefers to cleave short dsRNA with a guide strand possessing a 5′ phosphorylated A or U and a complementary strand harboring a 1-nt 3′ overhang, which measures 24 nt in length from the 5′ end of the guide strand (9, 96, 147). Therefore, Pol IV RNA featuring a 5′phosphorylated A primarily serves as the guide strand, while the RDR2 RNA featuring a 3′ 1-nt nontemplated overhang acts as the complementary strand during dicing by DCL3 (30, 87, 117). However, a smaller population of Pol IV RNAs are initiated with a 5′ phosphorylated G (9, 147). In this case, the RDR2 RNA may also have a chance to serve as a guide strand, because it initiates 1 or 2 nt internally to the 3′ end of the Pol IV RNA to yield 3′ overhangs at the Pol IV RNA, and DCL3 can tolerate guide strand RNA possessing a 5′ triphosphate, which is a feature of RDR2 RNA distinct from Pol IV RNA (15, 87). Overall, the short (∼26–45-bp) dsRNAs produced by Pol IV–RDR2 with a 5′ phosphorylated A and a 1-nt 3′ overhang are directly and selectively processed by DCL3.

The cryo-EM structure of DCL3 in complex with a precursor dsRNA in dicing-competent conformation uncovered the molecular basis of the specific dicing by DCL3 (132). DCL3 uses its PIWI–Argonaute–Zwille (PAZ) domain to split the first base pair of the precursor dsRNA, with the 5′ phosphorylated A of the guide strand, that is, the Pol IV strand, being flipped-out and specifically recognized (132). The splitting action of the first base pair also explains why a 5′ phosphorylated U is second after A in terms of preference (96), as an A–U pair is easier to split than a G–C pair. The 1-nt overhang allows the 3′ end of the complementary strand, that is, the RDR2 strand, to precisely approach and interact with several aromatic residues of the DCL3 PAZ domain (132). Therefore, DCL3 specifically recognizes the key end features of Pol IV–RDR2-produced dsRNA to ensure that only the Pol IV–RDR2 RNA, and not other RNAs, is processed. By using the 5′ end of the guide strand as an anchoring site, DCL3 measures 24 nt along the guide strand and cleaves the guide and complementary RNA strands with its second and first RNase III domains, respectively (132). The relative position of the two RNase III domains produces a 2-nt 3′ overhang at the guide strand, resulting in an asymmetric dsRNA product: a 24-nt RNA product derived from the Pol IV strand and a 23-nt RNA from the RDR2 strand (132). The 24-nt Pol IV strand is loaded into AGO4 as a guide strand with the 23-nt RDR2 strand as a passenger strand (130) (see the detailed discussion in Section 2.3). Therefore, the 24-nt siRNA responsible for RdDM is primarily shaped by a series of precisely connected events: the initiation of a 5′ phosphorylated A by Pol IV transcription, the addition of a 1-nt nontemplated 3′ overhang by RDR2, and terminus-specific dsRNA recognition and asymmetric dicing by DCL3.

The ribose 2′-OH group of the 3′ end nucleotide in plant sRNA is 2′-*O*-methylated by HUA ENHANCER 1 (HEN1), which protects it from further modifications and/or degradation (74, 144). Structurally, HEN1 requires an RNA substrate with a 2-nt 3′ overhang (48, 144). Therefore, only the Pol IV strand with a 2-nt 3′ overhang produced via DCL3 dicing undergoes methylation and is protected in principle and not the RDR2 strand with an only 1-nt 3′ overhang. Whether the 23-nt RDR2 strand is methylated needs to be confirmed, however (Figure 1). Despite the methylation of the 24-nt siRNA (74, 144), the *hen1* mutant does not show severe defects in RdDM (123), suggesting a dispensable role. Considering that RdDM requires Pol IV and Pol V to transcribe the same regions, the 24-nt siRNAs are likely used immediately upon production. Thus, protection by HEN1 may happen but not be essential. The precise role of HEN1 in RdDM, and especially its biological significance, needs further investigation.

### 2.3 RNA Polymerase V–Based Long Noncoding RNA Production and DNA Methylation

Downstream of RdDM and in parallel to the production of 24-nt siRNA by Pol IV, Pol V plays a central role in transcribing lncRNA to yield the Pol V–lncRNA complex, serving as a scaffold that recruits downstream DRM2 and gene silencing machinery (4, 8, 41, 93, 94, 136, 160) (Figure 1). The Pol V active site is conserved among DdRP family members and was shown to be essential for its functions in RdDM (38, 65), establishing a direct link between Pol V transcription activity and DNA methylation. However, unlike other DdRPs that mainly produce RNA transcripts, Pol V must accomplish its chromatin location function during dynamic transcription, suggesting unique enzymatic characteristics adapted to its scaffold function. Like Pol IV, Pol V has evolved from Pol II, and its largest subunit, NRPE1, is unique among all DdRPs, while its second-largest subunit, NRPE2, is identical to NRPD2 of Pol IV but different from the equivalent subunit of Pol II (37, 47, 65, 112, 141). Biochemically, Pol V transcription is slower but more faithful than Pol II (39, 89).

Although where and how Pol V transcription starts and ends remain unclear, recent structural studies of Pol V in its elongation conformation have revealed insights into the molecular mechanisms underlying its scaffold function (141, 142, 152). The structures of cauliflower (*Brassica oleracea* var. *botrytis*) and *Arabidopsis* Pol V revealed an overall architecture typical of that of eukaryotic DdRPs such as the canonical Pol II (18, 142, 152). The trigger loop element of the Pol V NRPE1 subunit is shorter and less flexible than that of Pol II, partially explaining the lower transcription rate of Pol V (142). A key to understanding the transcription features of Pol V lies in its NRPE2 subunit. First, a conserved tyrosine residue in NRPE2 clade members precisely stacks with the last base pair of downstream dsDNA to possibly prevent it from moving forward through stacking and hydrogen-bonding interactions, attenuating transcription elongation (142). In vitro biochemical assays confirmed that transcriptional arrest occurs when Pol V reaches dsDNA (142). Therefore, Pol V may transcribe slowly due to this blockage. Second, NRPE2 has a series of surface pockets that help capture the bases of the nontemplate strand ssDNA region within the transcription bubble to enhance Pol V backtracking (142). This enhanced backtracking of Pol V prevents it from moving too fast and allows it to oscillate back and forth around the target loci. Because DdRP backtracking triggers 3′ to 5′ cleavage, which is essential for removing the improperly paired base to support DdRP proofreading (97), the enhanced backtracking of Pol V explains its higher fidelity relative to that of other DdRPs (89). Overall, the Pol V NRPE2 subunit may slow down transcription and enhance backtracking, leading to Pol V retention at particular chromatin regions and allowing the timing for AGO4 and other effectors to deposit silencing marks. The biological consequence of the high fidelity of Pol V remains unclear. The structure of Pol IV–RDR2 indicates a possible cyclical production of precursor siRNA at particular loci to generate multiple siRNA copies (46). One lncRNA may therefore have multiple siRNA candidates with which to pair, necessitating higher accuracy for the lncRNA but less for the siRNAs. Alternatively, the high fidelity of Pol V lncRNA might be a simple biochemical consequence of its strong backtracking, without a precise biological function, as the AGO proteins that facilitate the recognition of the target RNA by its siRNA can withstand minor mismatches (134).

The connection between Pol IV–based 24-nt siRNAs and Pol V–based lncRNAs is AGO4 (161) (Figure 1). AGO4 captures the upstream siRNA produced by the Pol IV–RDR2–DCL3 module to base-pair with the lncRNA generated by Pol V; AGO4 also interacts with Pol V directly via the AGO-hook motif of the Pol V subunit NRPE1 and indirectly through the AGO-hook motif of Pol V–associated SUPPRESSOR OF TY INSERTION 5–LIKE (SPT5L), forming a higher-order complex that recruits DRM2 for de novo DNA methylation (8, 25, 28, 64, 72, 83, 135, 136, 154). SPT5L, a homolog of yeast SPT5 elongation factors, binds to AGO4, Pol V, and Pol V–produced lncRNA (8, 41, 115, 152). The AGO-hook motifs of SPT5L and Pol V NRPE1 function redundantly in recruiting AGO4 and DRM2 to Pol V (64). Structurally, a KOW (Kyprides, Ouzounis, Woese) domain of SPT5L binds close to the RNA exit channel of Pol V, in agreement with its role in recruiting AGO4 to bind to the lncRNA (152). Biochemically, AGO4 shows RNA-slicing activity in vitro and in vivo (109). In vivo, AGO4 predominately binds to 24-nt siRNA with a 5′ monophosphorylated A (40, 95, 109, 130, 131). However, recombinant purified AGO4 binds to a guide RNA without clear preference for RNA length or the 5′ end nucleotide, although it does prefer 5′ monophosphate over 5′ triphosphate, suggesting that the Pol IV–derived but not RDR2-derived 24-nt siRNA is preferentially selected by AGO4 (130). Notably, DCL3, AGO4, and Pol V, but not Pol IV, were detected in the same nuclear speckles, such as Cajal bodies (72, 106), suggesting a possible coupling between 24-nt siRNA production by DCL3, loading into AGO4, and docking of AGO4–siRNA with Pol V in certain speckles. The physical space constraints may contribute to concentrating DCL3-dependent 24-nt siRNA but not other siRNAs for loading into AGO4 in vivo. Additionally, according to the observation of abundant cytoplasmic 24-nt siRNA duplexes, an siRNA-shuttling mechanism was proposed (145). In this model, the DCL3-produced nascent 24-nt siRNA duplex is translocated from the nucleus into the cytoplasm, where it is loaded into the HEAT SHOCK PROTEIN 90 (HSP90)-chaperoned RNA-free AGO4 (145). The loading of siRNA induces the conformational change of AGO4 to release HSP90 and to expose AGO4’s nuclear localization signal for translocating back to the nucleus for RdDM (145). Further studies are required to unveil the precise siRNA delivery and loading mechanisms.

DCL3 produces an asymmetric siRNA duplex consisting of a 24-nt guide strand and a 23-nt passenger strand (87, 96, 130, 132). Upon loading into AGO4, the 23-nt passenger strand is sliced by AGO4 with the 3′ 11-nt fragment released and the 5′ 12-nt fragment retained, attaching to the 24-nt siRNA–AGO4 complex (130). The release of the 3′ 11-nt passenger fragment exposes the 5′ half of the guide RNA, known as the seed region, which initiates binding to the target RNA (32), making the siRNA–AGO4 complex competent to anneal to the target lncRNA (130). The base-pairing of the guide siRNA seed region with its target lncRNA may trigger the full annealing of the 3′ half of the guide siRNA to the target lncRNA to displace the 5′ 12-nt passenger fragment and then slice the target lncRNA. Supporting this model, the tenth nucleotide of the sliced lncRNA is always U, which presumably matches the first A of the AGO4-bound 24-nt siRNA (83, 130). AGO4 may remain with the sliced Pol V lncRNA, further directing DNA methylation (130). Although AGO4 can be coimmunoprecipitated by DRM2 (154), the molecular basis of the recruitment of DRM2 by AGO4 remains unclear and an outstanding question in the field.

DRM2 is the ortholog of mammalian DNA methyltransferase 3A (DNMT3A) and belongs to a group of plant-specific DNA methyltransferases (MTases) and shares all of their necessary functional motifs and overall folding, although the order of these motifs is different from that of the classical DNA MTases, such as DNMT3A (12, 13, 154). The structures of DRM2–substrate DNA complexes revealed a large DNA deformation induced by DRM2 binding, which leads to the preference of DRM2 for CHH DNA sequences over CG substrates (11, 14, 28, 129). In addition to the DNA MTase domain, the N terminus of DRM2 possesses three ubiquitin-associated (UBA) domains (13). Although the UBA domains are dispensable for its catalytic activity, mutating critical residues in the UBA domains of DRM2 resulted in a dramatic loss of DNA methylation in vivo, suggesting a critical but yet unidentified role for these UBA domains not related to catalysis (42, 154).

### 2.4 Targeting of Pol IV and Pol V to Chromatin

The locus-specific DNA methylation by RdDM centers the transcription of Pol IV and Pol V, and these two processes are precisely regulated to control both the methylation sites and levels. Two groups of factors have been identified to modulate the global targeting of Pol IV to chromatin: histone readers and the CLASSY (CLSY) family of chromatin remodelers (92). CLSY1, as well as its paralogs CLSY2–CLSY4, were shown to interact with Pol IV and regulate RdDM in both a locus- and a tissue-specific manner, with a *clsy1 clsy2 clsy3 clsy4* quadruple mutant showing a similar loss of 24-nt siRNA production as that of the *pol iv* mutant (69, 118, 143, 158, 159). The four CLSYs each control a subset of nonoverlapping 24-nt siRNA clusters, suggesting a locus-specific regulation of RdDM by each CLSY (159). A conserved motif in Pol IV was recently identified as the docking region that can accommodate a single CLSY, thus limiting each Pol IV to one CLSY binding at any given time, leading to a one CLSY per Pol IV model (29, 159). CLSY1 and CLSY2 synergistically regulate the production of 24-nt siRNA from chromosome arms in a histone 3 lysine 9 methylation (H3K9me)-dependent manner, while CLSY3 and CLSY4 regulate 24-nt siRNA production at pericentromeric heterochromatin in an H3K9me-independent manner (159) (Figure 1). Mechanistically, the *clsy1 clsy2* double mutant, but not the *clsy3 clsy4* double mutant, disrupts the association between Pol IV and SAWADEE HOMEODOMAIN HOMOLOG 1 (SHH1), a known H3K9me reader (67, 151, 159). More precisely, the *clsy1 clsy2* and *shh1* mutants display essentially the same decreased abundance of 24-nt siRNA clusters and CHH hypomethylated regions (159). SHH1 specifically recognizes unmethylated H3K4 and methylated H3K9 via its SAWADEE domain (67, 69, 151). Therefore, SHH1 may interact with CLSY1 or CLSY2 to recruit CLSY1- or CLSY2-bound Pol IV to H3K4me0K9me1/H3K4me0K9me2 regions, mediating H3K9me-dependent RdDM at chromatin arms (Figure 1). By contrast, the 24-nt siRNA loci controlled by CLSY3 and CLSY4 are independent of SHH1 and H3K9me but partially depend on CG methylation, with the underlying mechanism remaining unclear (159). ZINC FINGER, MOUSE DOUBLE-MINUTE/SWITCHING COMPLEX B, PLUS-3 PROTEIN (ZMP), a plant homeodomain (PHD) finger protein binding to unmethylated H3K4, was reported to associate with Pol IV and to regulate 24-nt siRNA production and DNA methylation at pericentromeric heterochromatin regions (133). The production of 24-nt siRNAs at ZMP-controlled regions was decreased in *clsy3 clsy4* but not in *clsy1 clsy2*, suggesting a possible link between ZMP and CLSY3/CLSY4 in RdDM of heterochromatin, offering a parallel to the SHH1–CLSY1/CLSY2 module in RdDM of euchromatin (92, 133) (Figure 1). Notably, CLSY3 is enriched at some loci with a highly conserved DNA motif (158). However, the underlying molecular mechanism needs further study.

The regulation of Pol V is much more complex. The putative sucrose nonfermenting 2 (SNF2)-type chromatin remodeler DEFECTIVE IN RNA-DIRECTED DNA METHYLATION 1 (DRD1), the structural maintenance of chromosomes (SMC) domain– containing protein DEFECTIVE IN MERISTEM SILENCING 3 (DMS3), and the putative methylated ssDNA–binding protein RNA-DIRECTED DNA METHYLATION 1 (RDM1) were shown to form a chromatin remodeling complex, termed the DDR complex, to regulate global Pol V occupancy in RdDM (31, 59, 61, 66, 155). Single mutants of all DDR components showed dramatically lower association of Pol V to chromatin, suggesting a critical role for the DDR complex (155). DRD1 directly interacts with the NRPE1 subunit of Pol V, and its chromatin remodeling function is required for efficient RdDM (59, 61, 66). A DRD1 peptide lacking its C-terminal helicase domain can form a complex with RDM1 and DMS3 with a stoichiometry of 1 (DRD1):2 (RDM1):4 (DMS3). This DDR subcomplex adopts a very compact structure, with an RDM1 dimer in the middle, flanked by two DMS dimers, and one DRD1 peptide wrapped asymmetrically around RDM1 and DMS3 (137). This DDR subcomplex is unique in its structure, potentially serving as a scaffold to recruit other effectors, while the DRD1 helicase domain might coordinate with Pol V for transcriptional regulation (137).

A known chromatin targeting mechanism of Pol V relies on SU(VAR)3–9 HOMOLOG 2 (SUVH2) and SUVH9 (56, 86). SUVH2 and SUVH9 contain su(var)3–9, enhancer of zeste and trithorax (SET) and SET and RING-associated (SRA) domains and are involved in RdDM and H3K9 methylation (57, 63, 103). While biochemical and structural studies have suggested that their SRA domains bind to methylated DNA, their SET domains appear to lack the post-SET subdomain and are likely to be catalytically inactive (56, 57). SUVH2 and SUVH9 directly interact with DMS3 in the DDR complex, thereby indirectly recruiting Pol V to methylated DNA (56, 86) (Figure 1). The *suvh2 suvh9* double mutant accumulated lower levels of both 24-nt siRNA and CHH methylation at Pol V–controlled methylation sites (56, 86). The regulation of Pol V occupancy by SUVH2 and SUVH9 in RdDM establishes a positive feedback loop for DNA methylation, potentially supporting the role of RdDM in CHH methylation maintenance (see the discussion in Section 3.2).

Notably, Pol IV and Pol V are both regulated by chromatin remodeler–epigenetic reader pairs. Pol IV possesses the chromatin remodeler partners of CLSYs and histone reader partners of SHH1 and ZMP (67, 92, 133, 151, 159). Pol V interacts with the chromatin remodeler DRD1 and the methylated DNA readers SUVH2 and SUVH9 (56, 86). The striking similarities between the regulation and targeting of Pol IV and Pol V suggest a plausible standard working procedure: A reader finds the target sites to which it recruits chromatin remodelers that relax the local chromatin architecture, after which the chromatin remodelers recruit Pol IV and Pol V for transcription. Many other factors, such as DRM3, SU(VAR)3–9–RELATED 2 (SUVR2), and JUMONJI 14 (JMJ14), to name a few, also regulate RdDM to some extent. Their precise roles and positions in RdDM and working mechanisms at the molecular level have not been thoroughly studied. The current understanding of all these factors has been extensively reviewed elsewhere (93, 94), and we do not discuss them in this review.

## 3 Maintenance of DNA Methylation in Plants

**Figure 2:**
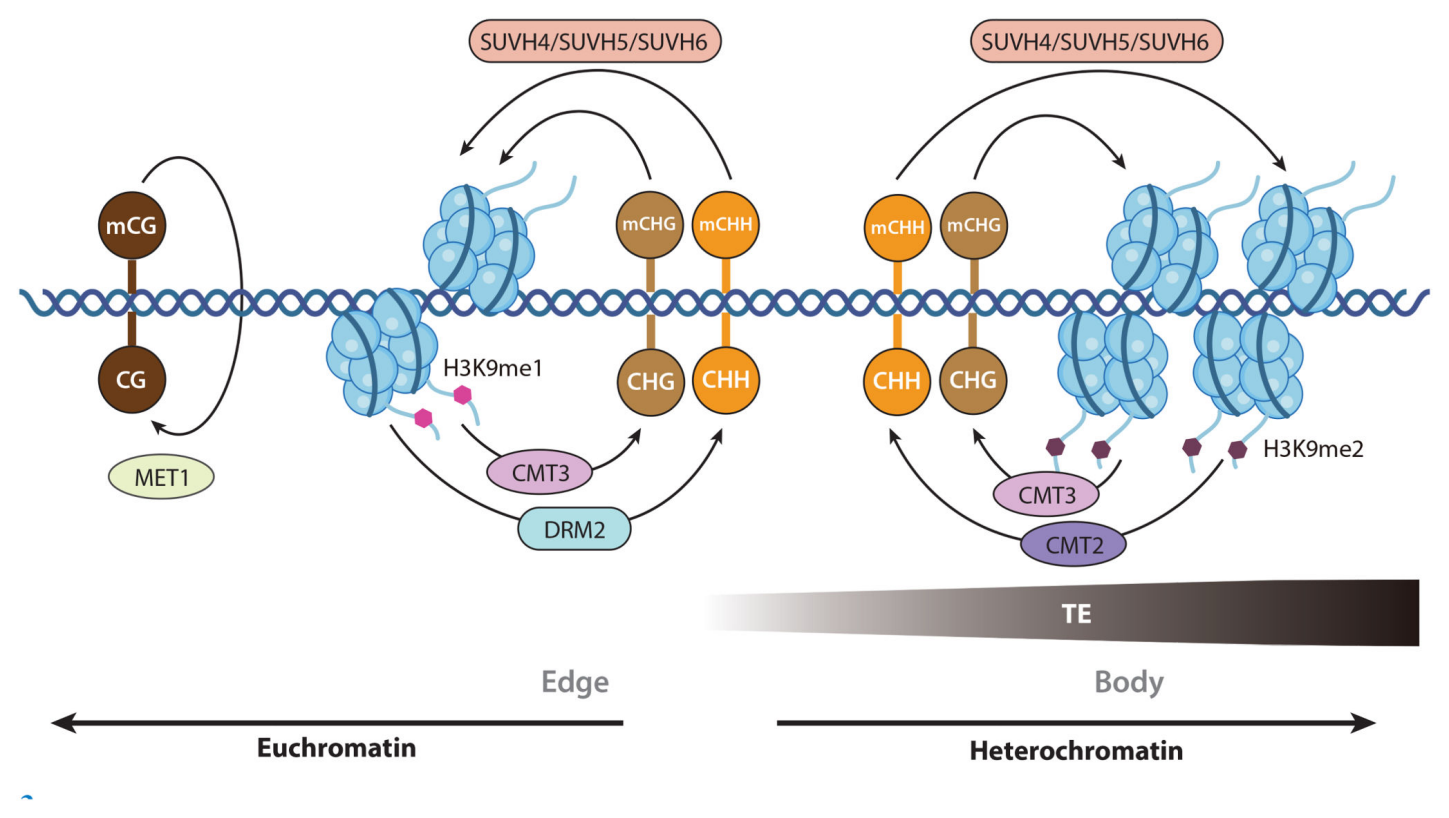
Maintenance DNA methylation in plants. CG methylation is maintained by MET1 via hemimethylated CGs. Non-CG methylation at TE regions is maintained via a self-reinforced loop between methylated DNA–directed H3K9 methylation by SUVH proteins and H3K9me-directed DNA methylation by CMT3 (CHG methylation), CMT2 (mainly CHH methylation over the TE body), and DRM2 via RdDM (mainly CHH methylation at short TEs and the edges of long TEs). Abbreviations: CMT, CHROMOMETHYLASE; DRM2, DOMAINS REARRANGED METHYLTRANSFERASE 2; H3K9me, histone 3 lysine 9 methylation; MET1, METHYLTRANSFERASE 1; RdDM, RNA-directed DNA methylation; SUVH, SU(VAR)3–9 HOMOLOG; TE, transposable element.

Once established, DNA methylation is relatively stable. However, during DNA replication, DNA synthesis only uses unmethylated nucleotides as substrates, yielding a dsDNA product comprising a methylated template strand and a fully unmethylated daughter strand. Therefore, DNA replication dilutes the preexisting DNA methylation by half, representing a decrease in DNA methylation. As it is critical for genome stability and multiple aspects of biology, DNA methylation must be reestablished on the daughter strand after DNA replication to maintain the methylation level across the genome. In plants, the maintenance of CG and non-CG methylation employs distinct pathways (68) (Figure 2).

### 3.1 The Maintenance of CG Methylation

Symmetric CG methylation yields a hemimethylated CG replication product with one strand retaining a residual methylated CG site, serving as a molecular mark to guide the maintenance of DNA methylation. In mammals, the maintenance DNA MTase DNA methyltransferase 1 (DNMT1) specifically recognizes hemimethylated CG DNA and methylates the newly synthesized unmethylated strand after the replication fork to faithfully maintain the CG methylation (34, 120). METHYLTRANSFERASE 1 (MET1), the plant ortholog of DNMT1, was identified as the CG maintenance MTase in *Arabidopsis* (58) (Figure 2). MET1 shares most functional domains with DNMT1, including the N-terminal replication focus targeting sequence (RFTS) domain, two adjacent bromo-adjacent homology (BAH) domains, and the DNA MTase domain. However, MET1 lacks the unmethylated CG DNA–binding CXXC domain of DNMT1. The RFTS and CXXC domains of DNMT1 autoinhibit the MTase domain to prevent the de novo methylation of unmethylated CG sites (119, 124). Moreover, the RFTS domain and the first BAH domain of DNMT1 recognize H3K9me3 and ubiquitylated H3 (RFTS), or H4K20me3 marks (BAH), to achieve histone modification–based regulation of DNMT1 (49, 113, 114). The absence of the CXXC domain suggests that MET1 may no longer show autoinhibition triggered by unmethylated CG DNA. In plants, gbM has not been reported to be associated with histone modification, and the *suvh4 suvh5 suvh6* triple mutant eliminating H3K9me2 modification has little effect on CG methylation (6, 123). Therefore, it is likely that MET1 is not regulated by H3K9me2, a histone mark highly associated with DNA methylation in plants (see the discussion in Section 3.2).

Several cofactors were reported to be associated with MET1 in regulating CG methylation. The VARIANT IN METHYLATION (VIM) family of methylated DNA–binding proteins are orthologs of the DNMT1 cofactor ubiquitin-like with PHD and RING finger domains 1 (UHRF1) (138). The *vim1 vim2 vim3* triple mutant showed dramatically lower levels of CG and CHG methylation, and the methylation levels of the VIM binding targets are reduced significantly in the *met1* mutant (62, 123). The plant HISTONE DEACETYLASE 6 (HDA6) can directly interact with MET1, and the *hda6* mutant showed loss of CG methylation at certain chromatin regions, suggesting a role for HDA6 in MET1-dependent CG methylation (24, 81, 84, 127). Mechanistically, MET1 may require the erasure of active histone acetylation marks by HDA6 before methylating the daughter strand, suggesting a coupling of these two epigenetic modification states (10).

So far, structural investigations into MET1-mediated plant CG methylation maintenance are lacking. Despite the similarities, plant MET1 has multiple features distinct from DNMT1 at the enzymatic and regulatory levels. Further biochemical and structural studies are required to reveal the molecular mechanism underpinning plant CG methylation maintenance.

### 3.2 The Maintenance of Non-CG Methylation

Unlike symmetric CG methylation, CHG is incompletely symmetric, and CHH is always asymmetric. Therefore, the replication products of all CHH methylation and some CHG methylation lack the necessary template strand to remember the proper methylation status in contrast to CG methylation, demanding external factors to deliver adequate signals. In plants, this signal is H3K9 methylation (Figure 2). The crosstalk between DNA and H3K9 methylation was discovered from a suppressor screen of the epiallele *clark kent*, caused by non-CG hypermethylation at the *Arabidopsis SUPERMAN* locus (52). While mutations in the DNA MTase gene *CHROMOMETHYLASE 3* (*CMT3*) eliminated the CHG hypermethylation at the *SUPERMAN* locus in the epiallele *clark kent*, mutations in the gene *KRYPTONITE* (*KYP*; also reported as *SUVH4*) encoding a SET domain–containing H3K9 MTase surprisingly resulted in the same phenotype as *cmt3*, diminishing CHG methylation (51, 79). Similarly, CMT3 and the SUVH4 paralog SUVH6 were reported to be essential for non-CG methylation of reporter constructs harboring the *PHOSPHORIBOSYLANTHRANILATE ISOMERASE* promoter (5, 88). DNA MTase CMT3 carries a chromodomain that recognizes the repressive histone mark H3K9me2, while the H3K9 MTase SUVH4 and its paralogs SUVH5 and SUVH6 harbor an SRA domain that recognizes methylated DNA, indicating a positive feedback loop between DNA and H3K9 methylation by CMT3 and SUVH4–SUVH6 (20, 50, 55, 68, 80, 110).

Mechanistically, CMT3 shows a preference in vitro for CHG methylation activity, and the structure of a CMT3 ortholog from maize (*Zea mays*), Zea methyltransferase 2 (ZMET2), in complex with the H3K9me2 peptide, revealed that the chromodomain and BAH domain of ZMET2 serve as the H3K9me2 reader that recruits the enzyme to H3K9me2-marked nucleosomes and facilitates H3K9me2-guided CHG methylation (21). A follow-up cryo-EM study suggested that ZMET2 prefers dinucleosomes as its substrate to methylate the linker but not nucleosomal DNA through a nucleosome-bridging mechanism (121). More precisely, the chromodomain mainly contributes to the stable binding of ZMET2 to H3K9me2, while the BAH domain facilitates binding to H3K9me2 in cooperation with binding to H3K18 in the same tail, inducing the repositioning of an allosteric loop within ZMET2 to create an additional DNA-binding interface for stimulating its activity (27, 121). CMT3 is a primary CHG maintenance MTase whose substrate DNA carries a to-be-methylated C on the daughter strand 2 nt downstream of the 5mC on the template strand. The crystal structure of ZMET2 in complex with hemimethylated CAG DNA showed that the to-be-methylated C on the daughter strand is flipped-out and that it fits into the catalytic pocket of ZMET2, while 5mC at the template strand is recognized specifically by a hydrophobic concavity of ZMET2 (27).

CHH methylation is maintained mainly by CMT2, a paralog of CMT3, in cooperation with the RdDM pathway discussed above (122, 146). CMT2 has a similar domain architecture as CMT3 but possesses an extra long N-terminal disordered region to mediate the heat-induced protein degradation, implying a role in connecting environmental stress response and DNA methylation (54). CMT2 binds to H3K9me and therefore was proposed to act like CMT3 to methylate the CHH site under the guidance of H3K9me modification (122). Moreover, the H3K9me reader SHH1 guides the RdDM pathway to H3K9me-marked loci for CHH methylation maintenance (67, 151). Unlike SHH1 and CMT3, however, which bind to H3K9me1, H3K9me2, and H3K9me3 equally well, CMT2 has a strong preference for H3K9me2 and weaker binding to H3K9me1 and H3K9me3 in vitro (67, 122). Therefore, CMT2 and DRM2 in the RdDM pathway show different preferences for the location of CHH methylation. While CMT2 is primarily responsible for CHH methylation at the H3K9me2-enriched bodies of long TEs, DRM2 is responsible for CHH methylation at H3K9me1-enriched short TEs and the boundaries of long TEs (Figure 2), suggesting that different H3K9 methylation states mark distinct positions within TEs to be methylated by different pathways (122, 123, 146). A potential underlying mechanism for this differential H3K9 distribution might rely on the fact that long TE bodies enriched in H3K9me2 and located in heterochromatin are less prone to being transcribed, thus presenting a challenge for Pol IV and Pol V to transcribe over these loci and engage RdDM for DNA methylation. By contrast, short TEs and the boundaries of long TEs adjacent to active euchromatin regions with less-compacted chromatin structure and lower H3K9 methylation levels may not be sufficient to trigger the H3K9me2-dependent CMT2 methylation feedback loop but would allow Pol IV and Pol V transcription to initiate RdDM. Alternatively, considering the lower total CHH methylation levels (1.7%) compared to CG (24%) and CHG (6.7%) (17), it may be hard to start the mCHH–H3K9me self-reinforced feedback loop solely via CMT2 at the TE boundaries. The de novo activity of RdDM may in part supplement the CHH methylation here. Nevertheless, all these hypotheses require further experimental testing.

In plants, the H3K9 methylation mark is mainly deposited by the SUVH family H3K9 MTases SUVH4–SUVH6, with some minor contributions from SUVR proteins (122, 123). Here, we focus on the extensively studied SUVH proteins. The structure of SUVH4 in complex with methylated DNA and an H3 peptide substrate uncovered an architecture consisting of an SRA domain bound to methylated DNA and a pre-SET/SET/post-SET catalytic cassette flanked by a two-helix bundle (19). While the SUVH4 SRA domain recognizes methylated CHG or methylated CHH on one side, its pre-SET/SET/post-SET catalytic cassette captures the unmodified H3K9 and methyl-donor *S*-adenosylmethionine on the other side, directly connecting methylated DNA and H3K9 methylation (19). SUVH5 and SUVH6 adopt domain architectures and functional mechanisms similar to those of SUVH4 (76, 110). These three SUVH proteins redundantly mediate most H3K9me2 deposition, as the *suvh4 suvh5 suvh6* triple mutant almost eliminates H3K9 methylation and non-CG methylation (122, 123). Conversely, the *drm1 drm2 cmt2 cmt3* quadruple mutant showed the same dual defects in non-CG and H3K9 methylation (122, 123), reinforcing the idea that a feedback loop exists between these marks. Notably, the DNA–H3K9 methylation feedback loop appears to regulate the DNA methylation level in a sequence context–dependent manner. In *Arabidopsis*, CHG methylation in the CWG context (with W referring to A or T) is significantly higher than CCG methylation (17, 36, 82). While SUVH6 has no sequence preference, SUVH4 and SUVH5 prefer to bind to CWG methylation and methylated CG–containing CCG methylation sites, respectively (76). Therefore, the dominant H3K9 MTase SUVH4 may bring more H3K9me to CWG sites than SUVH5 does to its preferred CCG sites, which would lead to the greater recruitment of CMT3 to CWG sites and result in a higher level of DNA methylation, reflecting the precise and strict correspondence between DNA and H3K9 methylation (76).

### 3.3 The Crosstalk and Regulation of Maintenance DNA Methylation

The CG gbM is solely maintained by MET1 in euchromatins apart from H3K9me and non-CG methylation–enriched heterochromatin (6), thereby possessing less crosstalk with others. By contrast, there are extensive cross-activity for the enzymes and crosstalk among the DNA methylations in TE regions. Most plant DNA MTases have sequence preferences but also possess activities toward less-favored sequences (122). The *cmt2 cmt3* double mutant showed a more pronounced loss of CHG methylation than the *cmt3* single mutant, although the *cmt3* mutant already showed a substantial loss of CHG methylation (122, 123, 146). Similarly, the *drm1 drm2* double mutant, which blocks RdDM, showed decreased CHG methylation, while the *drm1 drm2 cmt3* triple mutant almost completely eliminated CHG methylation (11, 122). The *drm1 drm2 cmt2 cmt3* quadruple mutant resembled the *drm1 drm2 cmt2* triple mutant in terms of its CHH methylation levels, suggesting that CMT2 and RdDM/DRM2 but not CMT3 are primarily associated with CHH methylation (122). The CG methylation–deficient *met1* mutant showed loss of heterochromatic CHG methylation, overlapping with the CHG regions controlled by SUVH4–SUVH6, suggesting an H3K9me2-mediated effect (123). The non-CG methylation mutants, including *suvh4 suvh5 suvh6, cmt3*, and *drm1 drm2*, exhibited a loss of heterochromatic CG methylation as well (123).

Overall, CG methylation of euchromatin appears to be solely maintained by the presumed hemimethylated CG-dependent methylation by MET1, like DNMT1 (Figure 2). In heterochromatin, CMT2, CMT3, and DRM2 may inevitably cross-methylate less-preferred sites (21, 28, 122, 129). More substantially, heterochromatic DNA methylations may trigger H3K9 methylation through SUVH4–SUVH6 and subsequently mediate DNA methylation, leading to crosstalk among the three sequence contexts through H3K9me (Figure 2). Therefore, the maintenance of DNA methylation at heterochromatin is quite complex, with extensive cross-methylation and crosstalk in both direct and indirect manners.

Unlike relaxed euchromatin, which is open to DNA MTases, a critical challenge for highly condensed heterochromatin is the accessibility of DNA MTases to DNA. DECREASE IN DNA METHYLATION 1 (DDM1), a SNF2-family chromatin remodeler, resolves this problem. A forward genetic screen identified *DDM1* as a master regulator of plant DNA methylation to suppress TEs (128). The *ddm1* mutant has a decreased level of heterochromatic DNA methylation in all sequence contexts, which is consistent with the role of DDM1 in increasing chromatin accessibility, facilitating access of DNA MTases to heterochromatin (123, 146, 156). Several studies have recently suggested that DDM1 also contributes to replacing the active histone variant H3.3 with repressive H3.1 and depositing the repressive variant H2A.W (53, 71, 101, 157). The structure of DDM1 in complex with nucleosomes containing H2A.W and H3.3 did not indicate variant-specific interactions (71, 102, 149), suggesting dynamic interactions during histone variant exchange but not chromatin remodeling. A parallel study captured the DDM1–nucleosome complex in different nucleotide-bound (ADP, ATP analog, and nucleotide-free) conformations (85). In the nucleotide-free state, DDM1 clamps on one side of the nucleosome and stretches the nucleosomal DNA from the entry side, forming a bulge; upon ATP binding, DDM1 undergoes a conformational change to release the nucleosomal DNA bulge, allowing the DNA to slide to the exit side, and the hydrolysis of ATP to ADP converts DDM1 to its ADP-binding conformation, which resembles the nucleotide-free conformation and stretches the nucleosomal DNA from the entry side again (85). The exchange between ATP and ADP repeats this cycle to continuously pump nucleosomal DNA from the entry to the exit sides. A remaining question needing further study is how to target DDM1 to heterochromatin marked by DNA methylation and H3K9me and with a condensed chromatin structure.

## 4 DNA Demethylation in Plants

The robust DNA methylation system of plants, especially the positive feedback loop between DNA methylation and H3K9 methylation, potentially spreads methylation marks across the genome; this requires an antagonist system, namely the DNA demethylation system, to prevent DNA methylation from spreading to active genes (125, 126). Biochemically, an existing 5mC can be lost by insufficient DNA methylation recovery during DNA replication, termed passive DNA demethylation, or can be enzymatically converted to a regular cytosine, termed active DNA demethylation (148). Active DNA demethylation, one of the focal points of this review, dynamically regulates gene activity and plays critical roles in multiple biological functions (148). Active DNA demethylation, which is broadly shared by animals and plants, is presently understood to be based on the removal of 5mC to yield a single-nucleotide nick, which is then filled with a regular cytosine through base excision repair (BER) (116, 148). However, plant DNA demethylation presents distinct features.

**Figure 3:**
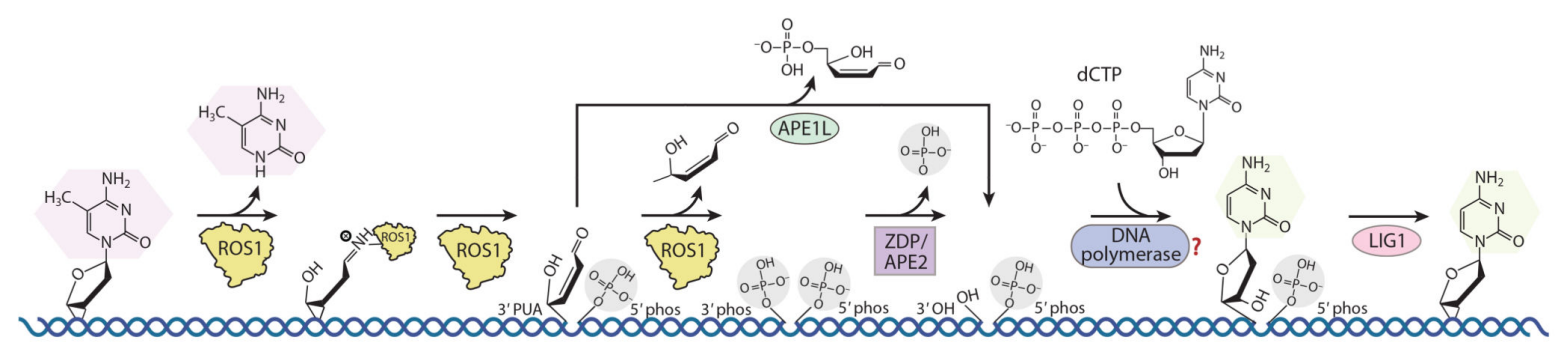
DNA demethylation in plants. Plant DNA demethylation is mediated by ROS1, a DNA glycosylase/lyase bifunctional enzyme that excises the 5mC base and incises the deoxyribose ring via a β-elimination reaction to produce 3′ PUA and 5′ phosphate. ROS1 can further mediate a δ-elimination reaction to convert 3′ PUA to a 3′ phosphate group. 3′ PUA and the 3′ phosphate groups are subsequently removed by APE1L and ZDP/APE2 to produce a 3′-OH group, which is then filled by a DNA polymerase to a regular cytosine. Finally, the ligase LIG1 seals the nick, resulting in DNA with an unmethylated cytosine. The involvement of a DNA polymerase has been proposed, but its identity remains unclear and is therefore indicated with a question mark. Abbreviations: 5mC, 5-methylcytosine; APE1L, APURINIC/APYRIMIDINIC ENDONUCLEASE 1–LIKE; APE2, APURINIC/APYRIMIDINIC ENDONUCLEASE 2; dCTP, deoxycytidine triphosphate; LIG1, DNA LIGASE 1; phos, phosphate; PUA, phospho-α,β-unsaturated aldehyde; ROS1, REPRESSOR OF SILENCING 1; ZDP, ZINC FINGER DNA 3′ PHOSPHOESTERASE.

In animals, the ten-eleven translocation (TET) family of dioxygenases successively oxidizes 5mC to 5-hydroxymethyl-, 5-formyl-, and 5-carboxyl-cytosine, with the latter two products recognized and excised by thymine-DNA glycosylase (TDG) to produce an apurinic/apyrimidinic (AP) site, which is repaired by the BER pathway (116). Plants have evolved a more robust system with a DNA glycosylase/lyase bifunctional enzyme REPRESSOR OF SILENCING 1 (ROS1), or its paralogs DEMETER (DME), DME-LIKE 2 (DML2), or DML3, to directly excise the 5mC base and subsequently incise the deoxyribose, yielding an ssDNA break in one step (2, 33, 35, 100) (Figure 3). While ROS1 has a lysine-rich N-terminal domain that enhances its DNA binding and potentially allows the enzyme to slide along DNA to search for the 5mC sites (104, 105), its C-terminal catalytic region possesses a glycosylase domain containing a 4Fe-4S cluster and an essential carboxy-terminal domain (CTD) of unknown function (45). The ROS1 family enzymes conservatively possess two long intrinsically disordered regions (IDRs) located within the glycosylase domain and between the glycosylase domain and CTD, respectively. The structure of the ROS1 catalytic fragment in complex with a methylated DNA substrate revealed a BAH domain fold for the CTD (23). The glycosylase domain of ROS1 adopts a canonical glycosylase fold with the 5mC base being flipped-out and captured by the base binding pocket formed by the glycosylase and BAH domains of ROS1 (23). The Watson–Crick edge and 5-methyl group of 5mC and the orphaned guanine followed by the flipping-out of 5mC are all strictly recognized by hydrogen bonds and hydrophobic interactions (23). Subsequently, a conserved lysine residue in the glycosylase domain approaches and attacks the deoxyribose ring to excise the base, resulting in an unstable Schiff base intermediate, which then undergoes β-elimination to yield an ssDNA break with a 3′ phospho-α,β-unsaturated aldehyde (PUA) and a 5′ phosphate and subsequent δ-elimination to convert the 3′ PUA to a 3′ phosphate (33) (Figure 3). The 3′ PUA and 3′ phosphate are cleaned by the AP endonucleases APURINIC/APYRIMIDINIC ENDONUCLEASE 1–LIKE (APE1L) and the 3′ DNA phosphatases ZINC FINGER DNA 3′ PHOSPHOESTERASE (ZDP) or APE2, respectively, to expose the 3′-OH group (70, 73, 77, 91) (Figure 3). In principle, an unknown DNA polymerase may add a regular cytosine nucleotide to fill the gap (Figure 3). Finally, the DNA LIGASE 1 (LIG1) seals the nick (78), yielding a regular C:G pair at the demethylated site (Figure 3).

The ROS1 family of DNA demethylases introduces ssDNA breaks at each DNA demethylation site, which would be harmful to the genome and must therefore be promptly repaired. Thus, ROS1 should be closely coupled with the downstream DNA repair machinery. The scaffold protein X-RAY CROSS-COMPLEMENTING PROTEIN 1 (XRCC1) interacts with ROS1 and ZDP to synergize their activities (90), suggesting that a scaffold-mediated protein–protein interaction may help couple these enzymes. Moreover, ROS1, APE1L, ZDP, and LIG1 colocalize in plant cells (77, 78, 91). Considering the two conserved IDRs of ROS1 family enzymes, phase separation or the formation of certain speckles may potentially contribute to tethering enzymes from the entire pathway together for efficient demethylation and prevention of DNA damage, although this hypothesis requires further investigation. Several protein complexes, such as the INCREASED DNA METHYLATION (IDM) histone acetyltransferase complex, the SWI2/SNF2-RELATED 1 (SWR1) chromatin remodeling complex, and the ROS1-ASSOCIATED WD40 DOMAIN–CONTAINING PROTEIN (RWD40) complex, are known to regulate ROS1 through unclear mechanisms (148). These complexes and their relationship to DNA demethylation have been precisely reviewed recently (148); we do not discuss them here.

## 5 Conclusions

In the past 30 years, powerful genetic studies have identified numerous essential factors mediating plant DNA methylation, assembling an almost complete image of the DNA methylation network in plants. Generally, plant DNA methylation is a very complex system that also involves multiple genetic and epigenetic regulatory pathways, with noncoding RNAs, histone modifications, and DNA repair playing critical roles in the establishment, maintenance, and removal of DNA methylation. Studies at the molecular level are the key to understanding the basic principles of DNA methylation in plants. The enzymatic features, specific interactions, subcellular localization, and even the accumulation of proteins into certain speckles likely cooperatively shape the initiation, maturation, and transduction of the signals that precisely mediate DNA methylation dynamics.

Although recent biochemical and structural studies have provided mechanistic insights, there are still many gaps in our understanding of DNA methylation in plants, underscoring the need for further biochemical and structural studies to fully uncover the molecular mechanisms underlying the establishment, maintenance, and removal of DNA methylation in plants.

## Figures and Tables

**Table 1:** Core components of plant DNA methylation pathways and their associated structures

Pathway	Protein	Full name	Short description	Protein Data Bank identifiers^b^	Reference(s)^c^
De novo DNA methylation	Pol IV^a^	RNA polymerase IV	RNA polymerase that produces single-stranded RNA (ssRNA) for the 24-nt small interfering (siRNA) precursor	7EU0,7EU1; 8XMB, 8XMC, 8XMD, 8XME	26, 46
	CLSY1–4	CLASSY 1–4	Chromatin remodelers that facilitate locus-specific 24-nt siRNA production	Not applicable (NA)	NA
	SHH1,DTF1	SAWADEE HOMEODOMAIN HOMOLOG 1,also reported as DNA-BINDING TRANSCRIPTION FACTOR 1	H3K9me-binding protein that regulates Pol IV occupancy	4IUP, 4IUQ, 4IUR, 4IUT, 4IUU, 4IUV	67
	ZMP	ZINC FINGER, MOUSE DOUBLE-MINUTE/SWITCHING COMPLEX B, PLUS-3 PROTEIN	Binds to unmethylated H3K4 (H3K4me0) and regulates Pol IV occupancy	NA	NA
	RDR2	RNA-DEPENDENT RNA POLYMERASE 2	RNA-dependent RNA polymerase that converts Pol IV RNA to dsRNA	7EU0, 7EU1; 7ROZ, 7RQS; 7W82, 7W84, 7W88 (*Zea mays*); 8XMB, 8XMC, 8XMD, 8XME	22, 26, 30, 46
	DCL3	DICER-LIKE 3	Dicer endonuclease that cuts Pol IV-RDR2-produced dsRNA to 23-24 nt	7VG2, 7VG3	132
	HEN1	HUA ENHANCER 1	SmallRNA(sRNA)3′ end nucleotide 2′-*O*-methyltransferase (MTase)	3HTX (22-nt sRNA complex)	48
	Pol V	RNA Polymerase V	RNA polymerase that produces scaffold long noncoding RNA (lncRNA)	8HIL, 8HIM (*Brassica* *oleracea*) 8HYJ	142, 152
	DRD1	DEFECTIVE IN RNA-DIRECTED DNA METHYLATION 1	The chromatin remodeler DRD1, the SMC hinge domain protein DMS3, and RDM1 form the DDR complex to regulate Pol V occupancy	6OIT	137
	DMS3	DEFECTIVE IN MERISTEM SILENCING 3		6OIS, 6OIT	137
	RDM1	RNA-DIRECTED DNA METHYLATION 1		6OIS, 6OIT; 1VK5; 2Q3T; 3GAN	3, 137
	SUVH2/9	SU(VAR)3–9 HOMOLOG 2/9	Binds to methylated DNA and regulates Pol V occupancy	4NJ5 (SUVH9)	56
	SPT5L, KTF1	SUPPRESSOR OFTY INSERTION 5-LIKE, also reported as KOW DOMAIN-CONTAINING TRANSCRIPTION FACTOR 1	AGO-hook protein that may recruit AGO4	8HYJ	152
	AGO4/6/9	ARGONAUTE 4/6/9	Argonaute proteins that bind to 24-nt siRNA and Pol V-lncRNA for recruiting DRM2	NA	NA
	DRM2	DOMAINS REARRANGED METHYLTRANS- FERASE 2	De novo DNA MTase	4ONJ, 4ONQ (*Nicotiana tabacum*); 7L4C, 7L4F, 7L4H, 7L4K, 7L4M, 7L4N; 8T1U	14, 28, 154
Maintenance DNA methylation	MET1	METHYLTRANSFERASE 1	CG site maintenance DNAMTase	NA	NA
	DDM1	DECREASE IN DNA METHYLATION 1	Chromatin remodeler that regulates the maintenance of DNA methylation	7UX9; 8WH5, 8WH8, 8WH9, 8WHA; 8KCB, 8KCC; 8J90	71,85, 102, 149
	VIM1-3	VARIANT IN METHYLATION 1–3	SRA domain–containing proteins that regulate MET1	7DUF (VIM1)	1
	HDA6	HISTONE DEACETYLASE 6	Histone deacetylase that associates with and regulates MET1	NA	NA
	CMT3	CHROMOMETHYLASE 3	CHG site maintenance DNAMTase	4FSX, 4FT2,4FT4 (*Zea* *mays),* 7UBU (*Z.mays*)	21,27
	CMT2	CHROMOMETHYLASE 2	CHH site maintenance DNAMTase	NA	NA
	SUVH4–6, KYP	SU(VAR)3–9 HOMOLOG 4–6, SUVH4 is also reported as KRYPTONITE	H3K9 MTase that binds to methylated DNA	4QEN, 4QEO, 4QEP (SUVH4); 3Q0B, 3Q0C, 3Q0D, 3Q0F (SUVH5); 4YGI (SUVH5); 6A5K, 6A5M, 6A5N (SUVH6); 7XPK (*Oryza sativa* SUVH6)	19, 76,110, 111,153
DNA demethylation	ROS1	REPRESSOR OF SILENCING 1	Bifunctional DNA glycosylase/lyase enzyme that excises 5mC and incises the DNA backbone	7YHO, 7YHP, 7YHQ	23
	DME	DEMETER	Paralog of ROS1	NA	NA
	DML2	DEMETER-LIKE 2	Paralog of ROS1	NA	NA
	DML3	DEMETER-LIKE 3	Paralog of ROS1	NA	NA
	ZDP	ZINC FINGER DNA 3′ PHOSPHOESTERASE	DNA 3′ phosphatase that converts the 3′ phosphate to a 3′-OH group	NA	NA
	APE2	APURINIC/ APYRIMIDINIC ENDONUCLEASE 2	Weak DNA 3′ phosphatase that converts the 3′ phosphate to a 3′-OH group	NA	NA
	APE1L	APURINIC/ APYRIMIDINIC ENDONUCLEASE 1-LIKE	AP endonuclease to convert 3′-phosphor-α,β-unsaturated aldehyde to a 3′-OH group	NA	NA
	POL?	DNA polymerase (to be identified)	To-be-identified DNA polymerase to fill the demethylation site by unmethylated C	NA	NA
	LIG1	DNA LIGASE 1	DNA ligase that seals the nick between the newly added C and DNA	NA	NA
	XRCC1	X-RAY CROSS- COMPLEMENTING PROTEIN 1	Putative scaffold protein that interacts with ROS1 and ZDP	NA	NA

a The full names corresponding to the abbreviations listed in this table are given only at first mention in the main text.

b Protein Data Bank identifiers (PDB IDs) from different articles are separated by semicolons. Structures with proteins from plants other than *Arabidopsis* and other notes are indicated in parentheses.

c The references corresponding to the structures (PDB IDs) are listed here. The articles delving into the functional characterization of each protein are cited in the main text.

## Glossary

### Abbreviations

Heterochromatin: a highly compact and condensed form of chromatin that is rich in TEs and tends to display low gene expression levels
Euchromatin: a relatively loose form of chromatin where most expressed genes are located
Gene body methylation (gbM): refers to the CG DNA methylation of exons for some plant housekeeping genes whose precise function remains unclear
Cryogenic electron microscopy (cryoEM): a powerful structural biology technique using a transmission electron microscope to determine the structures of biological macromolecules under cryogenic conditions
Small RNA (sRNA): short regulatory RNAs with a length of around 19–30 nt, including microRNA, small interfering RNA, and PIWI-interacting RNA
Long noncoding RNA (lncRNA): noncoding RNAs possessing longer length, usually more than 200 nt
NUCLEAR RNA POLYMERASE D 1 (NRPD1): the nomenclature for eukaryotic DdRP subunits, whereby the letter and number correspond to polymerase type and subunit number, respectively
Secondary channel: a channel in the DdRP structure that serves as the route for NTP substrate and for accommodating the backtracked RNA
Backtracking: occasional process whereby transcription by a DdRP moves backward to release and cleave the 3′ end of a nascent transcript
AGO-hook: a protein motif with tryptophan-glycine (WG) or GW repeats that mediates interactions with AGO proteins; also known as the WG/GW-containing motif
Seed region: a region in the AGO-bound RNA, usually located in nucleotides 2–8, that is responsible for initiating the binding to the target RNA
Methyltransferase (MTase): an enzyme that transfers a methyl group to a target molecule
Su(var)3–9, enhancer-of-zeste and trithorax (SET): a protein domain initially characterized in *Drosophila* proteins Su(var)3–9, enhancer-of-zeste [E(z)], and trithorax (Trx) that functions as a histone MTase
Passive DNA demethylation: a type of DNA demethylation caused by insufficient DNA methylation after replication, leading to its dilution and eventual removal
Active DNA demethylation: a type of DNA demethylation mediated by the enzyme-catalyzed active removal of 5mC
Base excision repair (BER): a DNA repair pathway that removes the mutated nucleotide and fills the resulting gap with the correct (wild-type) nucleotide
Apurinic/apyrimidinic (AP) site: a DNA lesion possessing only the sugar-phosphate backbone and lacking the base; also known as an abasic site
Intrinsically disordered region (IDR): a protein region that cannot form stable tertiary structure, recently shown to associate with phase separation or the formation of protein condensates

## Summary Points

1. The establishment of DNA methylation in plants relies on the RNA-directed DNA methylation (RdDM) pathway based on the plant-specific RNA polymerase IV (Pol IV) and Pol V to produce small interfering RNA (siRNA) precursors and long noncoding RNAs (lncRNAs), respectively.
2. Pol IV, RNA-DEPENDENT RNA POLYMERASE 2 (RDR2), and DICER-LIKE 3 (DCL3) form a relay chain for 24-nt siRNA production based on their enzymatic features, which ensures the precise production of Pol IV–derived 24-nt siRNAs for RdDM.
3. Due to its biochemical properties, Pol V can undertake dynamic transcription to achieve chromatin retention for its scaffold function in RdDM.
4. Plant CG methylation is maintained by METHYLTRANSFERASE 1 (MET1), likely through a mechanism similar to that of mammalian DNA methyltransferase 1 (DNMT1), which uses replication-produced hemimethylated CG DNA as a guide and intermediate.
5. Plant non-CG methylation is maintained via the H3K9me-based repressive mark as an intermediate through a self-reinforced loop between DNA methylation and H3K9 methylation.
6. Plant DNA demethylation employs the REPRESSOR OF SILENCING 1 (ROS1) family of bifunctional DNA glycosylase/lyase enzymes to specifically remove the 5mC and subsequently fill the gap with a regular cytosine by DNA repair.

## Future Issues

1. Where and how does Pol IV precisely initiate transcription?
2. Where and how does Pol V initiate and terminate transcription?
3. What are the precise actions and mechanisms of ARGONAUTE 4 (AGO4) in connecting Pol IV-based 24-nt siRNA and the Pol V–lncRNA complex and in recruiting DOMAINS REARRANGED METHYLTRANSFERASE 2 (DRM2)?
4. What is the recruiting mechanism for MET1?
5. How is DECREASE IN DNA METHYLATION 1 (DDM1) specifically recruited to its target loci before DNA methylation?
6. How do ROS1 and its paralogs find their target sites?
7. Which DNA polymerase functions in active DNA demethylation?
8. How is ROS1 coupled with the downstream DNA repair enzymes to prevent genome instability induced by DNA breaks?
